# Latamoxef dosing regimen adjustments and pharmaceutical care in pediatrics

**DOI:** 10.3389/fped.2024.1302087

**Published:** 2024-02-01

**Authors:** Ailin Zhang, Meiling Zuo, Yuxuan Sun, Jingtao Chen, Liqin Zhu, Wei Liu

**Affiliations:** ^1^ First Central Clinical College, Tianjin Medical University, Tianjin, China; ^2^ Tianjin Key Laboratory of Retinal Functions and Diseases, Tianjin Branch of National Clinical Research Center for Ocular Diseases, Eye Institute and School of Optometry, Tianjin Medical University Eye Hospital, Tianjin, China; ^3^ School of Statistics and Data Science, Nankai University, Tianjin, China; ^4^ Department of Pharmacy, Tianjin First Central Hospital, Tianjin, China; ^5^ Tianjin Children's Hospital, Children's Hospital of Tianjin University, Tianjin, China

**Keywords:** latamoxef, pediatrics, dosing regimen adjustments, pharmaceutical care, pharmacokinetics, pharmacodynamics, adverse drug reactions

## Abstract

Latamoxef is a semi-synthetic, broad-spectrum oxacephem antibiotic used primarily to treat infectious diseases, but the adverse drug reactions, such as the risk of fatal bleeding, once caused physicians to use it less frequently. However, with the rise of antibiotic-resistant bacterial strains, latamoxef is being used again to treat infectious diseases, especially in pediatrics. The pharmacokinetic parameters of latamoxef are highly variable, given the changes in body composition, organ maturation, and development that occurs in pediatrics. Therefore, an appropriate dosing regimen is essential. Latamoxef dosing optimization in pediatrics should adequately account for current body weight, postnatal age, postmenstrual age, and different minimum inhibitory concentration (MIC) values. In addition, attention should also be paid to some of the adverse reactions associated with latamoxef, such as coagulation disorders and bleeding risks, disulfiram-like reactions, as well as hypersensitivity and anaphylactic shock. This review summarizes the dosing regimens and some key points of pharmaceutical care for latamoxef in pediatrics in order to provide a better reference for its application in clinical practice.

## 1 Introduction

As a semi-synthetic, broad-spectrum oxacephem antibiotic, latamoxef was first used to treat infectious diseases caused by gram-positive and gram-negative aerobic and anaerobic bacteria in adults, children, infants, and neonates (1). However, the adverse drug reactions, particularly fatal hemorrhages, have led to latamoxef being used far less frequently (2, 3). Recently, due to the rise of antibiotic-resistant strains, latamoxef has gained renewed interest and has become a popular option for pediatric antimicrobial therapy (4).

Although latamoxef has been widely used in pediatric infectious diseases, its dosing regimens do not clarify the frequency and dose of administration and therefore do not provide specific guidance for clinical treatment. The pharmacokinetic parameters of latamoxef are highly variable among pediatrics due to changes in body composition, organ maturity, and development (5–7). Therefore, an appropriate dosing regimen is essential. In addition, latamoxef-related adverse reactions also need to be taken into account, given the speciality of pediatric patients.

The aim of this review is to provide dosing selection regimens for neonates, infants, and children, and to provide several pharmaceutical care points when using latamoxef. We hope that this review will serve as a useful reference for pediatric latamoxef therapy worldwide.

## 2 Latamoxef pharmacokinetics and pharmacodynamics

### 2.1 Pharmacokinetics

Latamoxef is well tolerated after intramuscular or intravenous administration, but it is not absorbed after oral administration. In neonates, the peak serum concentrations are approximately 105 μg/ml after the first dose and 128 μg/ml in steady state after given latamoxef 50 mg/kg, q12h (8). In infants, the peak concentration are 29–75 μg/ml, 68–124 μg/ml, and 200–260 μg/ml at completion of 10–15 min intravenous infusion of 25, 50, and 100 mg/kg, respectively (9). In children, the mean serum levels reach peaks of 96.6 and 76.0 μg/ml in 15 min after intravenous injection of latamoxef 20 mg/kg and 10 mg/kg, respectively (10). The mean serum levels after intravenous drip infusion over a 1-h interval reach peaks of 71.4 and 39.8 μg/ml at the end of the infusion of latamoxef 20 and 10 mg/kg, respectively (10).

The apparent volume of distribution (Vd) is 515–537 ml/kg and 518 ml/kg after administration of a dose of 50 mg/kg in neonates (0–28 day) and infants (1–24 months), respectively (9). In children aged 2 months to 7.5 years, the Vd of latamoxef is 0.4 L/kg (1).

Latamoxef is hardly metabolized in the body. The calculated mean rates of plasma clearance (CL_plasma_) are 16–31 ml/min per 1.73 m^2^ in neonates and 137 ml/min per 1.73 m^2^ in infants aged 1–24 months (9). Renal clearance (CL_R_) accounts for 63%–74% of total CL_plasma_, and a large amount of latamoxef is eliminated through glomerular filtration (11, 12). In pediatrics, elimination half-life values are inversely correlated with gestational and chronological age. The elimination half-life is 6.2 h in neonates less than one week of age, 4.4 h in those one to four weeks and 1.6 h in infants 1–24 months of age (13). In children aged 2 months to 7.5 years, the elimination half-life is 1.8–2 h (14).

### 2.2 Pharmacodynamics

Latamoxef has excellent activity against gram-negative aerobic bacteria, particularly the Enterobacteriaceae, such as *Escherichia coli* (*E. coli*) and *Klebsiella pneumoniae* (*K. pneumoniae*). The minimum inhibitory concentration of latamoxef for 90% (MIC_90_) of *E. coli* ranged from 0.125 to 0.5 µg/ml; for *K. pneumoniae*, 90% of isolates are susceptible to a concentration of 0.5 µg/ml or less (1). *Pseudomonas aeruginosa* (*P. aeruginosa*) isolates are moderately sensitive to latamoxef, with 50% being inhibited by 8–32 µg/ml, and concentrations of 64 µg/ml or more are usually required to inhibit 90% of the strains (1). Latamoxef is less active than the cephalosporins against most gram-positive bacteria. The MIC_90_ of *Staphylococcus aureus* (*S. aureus*) strains ranged from 4 to 16 µg/ml. Latamoxef has similar activity against penicillin-resistant and -sensitive strains of *S. aureus*, but methicillin-resistant *S. aureus* is resistant to latamoxef (MIC_90_ > 64 µg/ml) (15–18). The MIC_90_ of latamoxef for *Streptococcus pneumoniae* (*S. pneumoniae*) and *Streptococcus pyogenes* (*S. pyogenes*) varies from 1 to 3 μg/ml (18). However, enterococci are resistant to latamoxef (MIC_90 _≥ 64 µg/ml).

## 3 Dosing optimization of latamoxef based on pharmacokinetics/pharmacodynamics in pediatrics

### 3.1 Dosing optimization in neonates and infants

Neonates and infants have greatly variable changes in body composition, organ maturity, and development. Hence, special consideration must be given to dosing regimens for both populations.

Population pharmacokinetics (PPK) is a new branch in the field of pharmacokinetic study in recent years. Combining the classical pharmacokinetic model with a population-based statistical model, PPK is used to characterize the pharmacokinetic distribution of a specific population.

Qi et al. found that latamoxef CL in neonates and infants was significantly influenced by current body weight, birth weight, and postnatal age (19). Kou et al. also showed that current body weight and postmenstrual age were identified as significant covariates on latamoxef CL, and current body weight was identified as a significant covariate on latamoxef Vd (20). This change in CL with age can be attributed to renal maturation. In intrauterine life, the glomerular filtration function is established, but it is insignificant. This function begins immediately after birth as the kidneys begin to regulate fluids, water, and electrolytes (21). Newborns have a glomerular filtration rate (GFR) of approximately 40 ml/min/1.73 m^2^, which reaches 66 ml/min/1.73 m^2^ by 2 weeks of age. At approximately 2 years of age, GFR reaches adult levels of 100–125 ml/min/1.73 m^2^ (22). Due to the positive correlation between CL and age, the latamoxef dosing regimen was initially considered only the age factor: 50 mg/kg, q12h in neonates aged 0–1 week, q8h in neonates aged 1–4 weeks, and q6h in infants (1).

However, considering age alone is not sufficient as treatment efficacy is also affected by different bacteria. Hence, dose selection should be based on a combination of pharmacokinetics and pharmacodynamics. As a typical time-dependent antimicrobial agent, the action of latamoxef is largely dependent on the percentage of time when the blood concentration is higher than the minimal inhibit concentration (fT > MIC) during the dose interval (23). Effective dosing regimens for time-dependent antibiotics require that serum drug concentrations exceed the MIC of the causative pathogen for at least 40%–50% of the dosing interval (23). The fT > MIC values for latamoxef are mostly 50%–70% (19, 24, 25).

Monte Carlo simulation combines pharmacokinetic and pharmacodynamic parameters as well as pathogen epidemiology to assess the efficacy of antimicrobial regimens against target strains to optimize empiric antimicrobial therapy (26). Probability of target attainment (PTA) is defined as the percentage of subjects who achieve pharmacokinetics/pharmacodynamics target values. A dosing regimen with a PTA ≥90% is a reasonable choice for empiric antimicrobial therapy.

For the initial dosing regimens in neonatal bacterial infectious diseases, Kou et al. studied 15 dosing regimens under 4 different MIC values (24). The results of PTA of 15 dosing regimens under 4 different MIC values is shown in Table 1. A PTA value ≥90% is reckoned to have a satisfactory effect on the specific MIC. The result also indicates that when 4 μg/ml ≤ MIC ≤ 8 μg/ml, there is a statistically difference between PTA at different administration frequencies at the same dosage (*P* < 0.05); when MIC ≤ 2 μg/ml, there is no statistically difference between PTA at different administration frequencies at the same dosage (*P* > 0.05). In neonatal population, the optimal initial dosing regimen for bacterial infectious disease should be followed by a lower dose and less administration frequency, while at the same time achieving bacteriostatic effects. Consequently, Kou et al. recommended 60 mg/kg/day q12h as the desirable initial administration regimen for neonatal infectious diseases.

**Table 1:** The PTA for different dosing regimens of latamoxef at different MIC values (24).

Dosing regimen		PTA				*P*	
Dosage	Frequence	MIC = 1 μg/ml	MIC = 2 μg/ml	MIC = 4 μg/ml	MIC = 8 μg/ml	MIC ≤ 2 μg/ml	4 μg/ml ≤ MIC ≤ 8 μg/ml
80 mg/kg/day	q12h	96.7	92.5	82.5	55.5	>0.05	<0.05
	q8h	99.6	98.8	96.3	82.9		
	q6h	99.9	99.8	98.9	93.6		
70 mg/kg/day	q12h	96.1	91.1	79.2	47.2	>0.05	<0.05
	q8h	99.5	98.5	94.6	77.3		
	q6h	99.9	99.7	98.5	90.5		
60 mg/kg/day	q12h	95.4	90.2	74.4	36.1	>0.05	<0.05
	q8h	99.4	98.2	92.9	68.1		
	q6h	99.9	99.6	97.8	84.5		
50 mg/kg/day	q12h	94.2	86.8	67.4	23.4	>0.05	<0.05
	q8h	99.2	97.5	89.7	51.9		
	q6h	99.9	99.4	96.5	72.4		
40 mg/kg/day	q12h	92.5	82.6	55.5	9.5	>0.05	<0.05
	q8h	98.8	96.3	82.9	29.1		
	q6h	99.8	98.9	93.6	49.4		

PTA, probability of target attainment; MIC, minimal inhibitory concentration.

In other study, Qi et al. pointed out that for neonates and infants, 30 mg/kg q12h is effective against *E. coli* and *K. pneumoniae* (MIC ≤ 1 μg/ml). While, a higher dose about 30 mg/kg q8h is required against *S. aureus* (MIC = 4 μg/ml) (19).

As for the effect of current body weight on CL, Wang et al. also provided a choice that latamoxef dosing regimens could be based on children's body surface area (BSA), since they found that BSA was also a significant covariate to CL and Vd (25). The results of dosing regimens according to BSA under different MIC values are presented in Table 2.

**Table 2:** Dosage regimens of latamoxef based on different BSA stratifications and different MIC values (25).

BSA group	MIC90 (μg/ml)			
	0.5	1	2	8
0.2–0.4 m^2^	50 mg, q12h	100 mg, q12h	150 mg, q12h	200 mg, q6h
0.41–0.6 m^2^	100 mg, q12h	200 mg, q12h	375 mg, q12h	475 mg, q6h
0.61–0.8 m^2^	200 mg, q12h	375 mg, q12h	400 mg, q8h	625 mg, q6h
0.81–1.0 m^2^	300 mg, q12h	550 mg, q12h	600 mg, q8h	950 mg, q6h
1.01–1.2 m^2^	500 mg, q12h	925 mg, q12h	900 mg, q8h	1,400 mg, q6h

BSA, body surface area; MIC, minimum inhibitory concentration.

Although there are several dosing regimens options, these regimens are based on simulation results and the efficacy of these regimens needs to be further validated in clinical practice. Yet there is something undeniable about these regimens that provides scientific guidance that contributes to the rational use of latamoxef in pediatrics.

### 3.2 Dosing optimization in children

For children, dosage is generally between 40 and 80 mg/kg/day, q6h or q8h, as GFR has reached adult levels. Dosing adjustments based on BSA and MIC values may also be made if necessary (Table 2).

The shortcoming of the current study is that there is no appropriate regimen for children with renal insufficiency. Although renal function plays a crucial role in latamoxef excretion, creatinine clearance (CL_cr_) is not well correlated with latamoxef CL. The reason for this may be that the number of samples was limited and most of the participants had normal kidney function. Larger samples are therefore needed for further studies or to use physiologically based pharmacokinetic models to predict dosing regimens in children with renal dysfunction.

Table 3 summarizes the key findings of the pharmacokinetics/pharmacodynamics study of latamoxef in pediatrics.

**Table 3:** The key findings of the pharmacokinetics/pharmacodynamics study of latamoxef in pediatrics.

Population	Key findings of the pharmacokinetics/pharmacodynamics study	Reference
Neonates aged 0–28 days	Current weight and postmenstrual age are identified as a significant covariate on latamoxef CL. Current weight is identified as a significant covariate on latamoxef Vd.	(20)
Neonates and infants with median (range) of postnatal age 8.0 (1.0–54.0) day	Current body weight, birth weight, and postnatal age are identified as significant covariates influencing latamoxef CL 30 mg/kg q12h is effective against *E. coli* and *K. pneumoniae* (MIC ≤ 1 μg/ml); 30 mg/kg q8h were required against *S. aureus* (MIC = 4 μg/ml)	(19)
Children under 18 years old	Body surface area (BSA) is identified as the most significant covariate to Vd and CL Specific recommendations are given for dosage adjustment in pediatric patients based on BSA	(25)
Neonates in ICU	Monte Carlo simulation is used to recommend “60 mg/kg/day q12h” as the initial administration regimen	(24)

Vd, the apparent volume of distribution; CL, clearance; *E. coli*, *Escherichia coli*; *K. pneumoniae*, *Klebsiella pneumoniae*; *S. aureus*, *Staphylococcus aureus*.

## 4 Latamoxef pharmaceutical care based on adverse drug reactions

### 4.1 Adverse drug reactions of latamoxef in adults

Latamoxef is generally well tolerated in adults. Local reactions (3%), hypersensitivity (3%), diarrhea (1%), eosinophilia (2.5%), and abnormalities in liver (3%) or renal (2%) function tests are the most commonly reported adverse drug reactions. Other adverse drug reactions such as coagulation disorders, antibiotic-associated colitis, and disulfiram-like reactions have also been reported (1). 3% of patients treated with intravenous or intramuscular latamoxef have reported local reactions such as phlebitis and pain. However, these reactions rarely necessitate discontinuities (27, 28). Hypersensitivity, which manifests in the form of rash and/or drug fever, occurs in about 3% of patients treated with latamoxef. This is the most common reason for stopping treatment. Approximately 4% of patients allergic to penicillin or cephalosporins will have a cross-allergic reaction to latamoxef (18, 29). Patients especially those with a type Ⅰ hypersensitivity reaction to penicillin should use latamoxef with caution. The bleeding risk is thought to be related to the administration of latamoxef. Patients who are seriously ill, debilitated, or malnourished, or with renal impairment, are more likely to develop the condition (30, 31). Abnormalities of liver function manifest as hepatic enzyme abnormalities, such as increased serum glutamic oxaloacetic or pyruvic transaminases or alkaline phosphatase (29). Abnormal results on renal function tests, such as elevated blood urea nitrogen and serum creatinine, as well as hematuria, pyuria, and albuminuria, have been reported in approximately 2% of patients. However, they are rarely thought to be drug-related (29, 32).

### 4.2 Pharmaceutical care of latamoxef in pediatrics

Although adverse drug reactions of latamoxef have been less frequently reported in pediatrics, there are still some aspects that need to be addressed.

#### 4.2.1 Coagulation disorders and bleeding risk

The most notable adverse drug reactions during treatment with latamoxef are coagulation disorders and bleeding risks, which are a major reason for limiting its clinical use. Brandstetter et al. reported that a fatal pulmonary hemorrhage occurred on the eighth day of latamoxef therapy, and the patient had a noticeable elevation of prothrombin time (PT) and partial thromboplastin time (PTT) (3). Joehl et al. reported that in eight patients with abdominal infections treated with latamoxef, six of them had prolonged template bleeding times, and two had clinically significant hemorrhage (epistaxis, hematuria, and rectal bleeding) during treatment (2). Zhang et al. reported that a 28-year-old man with tuberculosis and Crohn's disease developed severe thrombocytopenia with scattered purpura and petechiae on the extremities and trunk after treatment with latamoxef for pulmonary bacterial infection. Platelet counts recovered and hemorrhagic symptoms decreased after discontinuation of latamoxef (33). Zhu et al. reported that two very elderly patients developed hemorrhage, and coagulation tests showed a longer PT, activated partial thromboplastin time (APTT), and a high international normalized ratio (INR), which was responsible for using latamoxef (31). A systematic review and meta-analysis showed that hypoprothrombinemia and PT were significantly associated with NMTT-cephalosporins, whereas bleeding was not. Subgroup analyses revealed a significant association between latamoxef and hypoprothrombinemia (34).

As a third-generation cephalosporin, latamoxef contains an N-methylthiotetrazole (NMTT) side chain, which can interfere with vitamin K metabolism and therefore induce coagulation disorders and bleeding risks (30). In addition, latamoxef can also inhibit adenosine diphosphate (ADP)-induced platelet aggregation by perturbing the platelet membrane, making ADP receptors unavailable to the agonist, thus causing coagulation disorders (35).

Malnutrition is a risk factor for coagulation disorders (1, 30, 36). This is because malnutrition may result in decreased vitamin K intake, decreased gastrointestinal absorption of vitamin K, and antibiotic inhibition of the gastrointestinal flora that synthesizes vitamin K, all of which lead to vitamin K-dependent coagulation and hemoglobin reduction (36). In particular, pediatrics between the ages of 1 month and 1 year are at risk for developing coagulation disorders when prolonged use of latamoxef due to poor vitamin K content in human milk or improper weaning practices (36). Therefore, in addition to monitoring coagulation indicator, prophylactic doses of vitamin K are recommended for pediatric patients who are undernourished and who are being administered intravenously for extended periods of time. Prophylactic vitamin K is recommended upon initiation of therapy and every two weeks thereafter for the duration of treatment (37).

Previous studies reported that bleeding risk may occur in patients who use latamoxef and anticoagulants concomitantly (38, 39). To avoid an increased risk of bleeding, it is best not to combine latamoxef with anticoagulant drugs or antiplatelet agents.

#### 4.2.2 Disulfiram-like reactions

Certain prescription medications can cause adverse effects when consumed with alcohol or ethanol. Among these, disulfiram-like reactions are relatively serious. Symptoms of mild reactions include flushing and headaches, while moderate to severe reactions can lead to hypotension, dysrhythmia, and death (40). Second to metronidazole, cephalosporin antibiotics, especially those containing a methylthiotetrazole (MTT) substituent, are known to cause disulfiram-like reactions (41). Given that latamoxef contains an NMTT side chain, attention should also be paid to the disulfiram-like reactions.

Children generally do not have drinking behavior, and disulfiram-like reactions in children are rarely reported. However, medicinal preparations containing ethanol should be used with caution. As a commonly used excipient or solvent in drug preparations, ethanol is highly likely to cause disulfiram-like reactions when patients need to combine medications, especially when cephalosporins or nitroimidazoles are co-administered with a particular drug that uses ethanol as an excipient or solvent. Small et al. reported that an 8-year-old male was administered a dose of prednisolone elixir (5% ethanol by volume) after a ceftriaxone infusion and then suffered a mild but likely disulfiram-like reaction (41). Li et al. found that 13 kinds of drugs containing ethanol were involved in the 119 cases of disulfiram-like reactions, most of which were hydrocortisone injection (70 cases, 58.83%) and huoxiangzhengqi liquid (21 cases, 17.65%) (42). They also pointed out that ethanol-containing injections, oral preparations, and external preparations could all induce disulfiram-like reactions, and the highest incidence of 76.47% was observed in combination with injections (42). Medicinal preparations containing ethanol are listed in Table 4.

**Table 4:** Medicinal preparations containing ethanol (42).

Drug classification	Drug name	Ethanol content
Adreno-cortical hormones	Hydrocortisone injection	50%
Cardiovascular drugs	Deslanoside injection	10%
	Digoxin injection	10% (V/V)
	Nimodipine injection	20% (V/V)
	Nitroglycerin injection	Not in detail
	Diazepam injection	Not in detail
Antibacterials	Azithromycin dihydrochloride injection	Soluble containing ethanol
	Amphotericin B liposome for injection	Excipients containing anhydrous ethanol
	Voriconazole for injection	Specialized solvents containing ethanol
Immunosuppressant	Tacrolimus injection	Excipients containing anhydrous ethanol
	Ciclosporin injection	Excipients containing ethanol
Anti-tumor drug	Docetaxel injection	Excipients containing anhydrous ethanol
	Paclitaxel injection	Excipients containing anhydrous ethanol
Oral preparations of chinese medicines	Huo-Xiang-Zheng-Qi	40%–50%
	Shi-Di-Shui	60%–70%
	Compound glycyrrhiza oral solution	Liquorice fluid extract and paregoric

Co-administration of medications containing ethanol in excipients and the use of alcohol baths should be avoided during the treatment of latamoxef and for at least 5 days after completion of the treatment (42, 43). At the same time, awareness of the risk of disulfiram-like reactions should be raised. When patients experience nausea, abdominal discomfort, vomiting, headache, flushing, or tachycardia after receiving latamoxef, a thorough review of all medications they are taking, both prescriptions and over-the-counter medications, must be performed to identify whether the ethanol is present (44). Once a disulfiram-like reaction occurs, drugs under use must be discontinued immediately. Patients with mild symptoms may recover gradually, while those with severe reactions need prompt medical treatment. Dexamethasone can counteract the stress response, accelerate ethanol oxidation, and be used to treat disulfiram-like reactions by increasing acetaldehyde dehydrogenase activity, usually at a dose of 5–10 mg administered intravenously (45, 46). Antihistamines can also be given; vitamin C, vitamin B6, and coenzyme A can be administered intravenously for nutritional support; mesocarbamol or dopamine can be prescribed for decreased blood pressure; and metoprolol can be given for tachycardia (45).

#### 4.2.3 Hypersensitivity and anaphylactic shock

Hypersensitivity may occur in approximately 2%–3% of patients treated with latamoxef and consist of symptoms including rashes, occasional fever, and eosinophilia (1, 18, 47). This was the most common reason for stopping treatment (1). Approximately 4% of patients allergic to penicillin or cephalosporins would have a cross-allergic reaction to latamoxef (18, 29). Therefore, patients with penicillin allergy should use latamoxef with caution. The study by Fan showed that allergic shock often occurred in pediatrics aged 3–6 years old, and the top three drugs were cephalosporins, penicillins, and macrolides (48). Medication histories are simpler in children, making it harder to identify allergens based on allergy history as it is conducted in adults; moreover, younger pediatric patients do not know how to call for help when they develop abnormal symptoms, and the treatment can be overdue due to the delayed detection of allergic reactions, which can lead to serious consequences (49).

Rapid assessment and maintenance of the airways, breathing, and circulation are the first steps in treating anaphylaxis. In case of anaphylaxis, even if the symptoms do not involve vital organs, adrenaline or epinephrine must be administered immediately intramuscularly, 0.01 mg/kg in children with a maximum dose of 0.3 mg every 5–15 min, in an attempt to prevent more severe anaphylaxis (50). In the case of urticaria and angioedema, H1 antihistamines are the most effective medications. Second-generation H1 antihistamines are generally considered the optimal option in treatment, because of the lower sedative effects (51). Among patients with severe symptoms, particularly those with angioedema, a relatively brief course of systemic corticosteroids (0.5–1 mg/kg/day) may be considered for better symptom control (50).

## 5 Conclusions

Latamoxef has excellent antimicrobial properties against most pathogens, making it a popular option for pediatric antimicrobial therapy. Given that body composition changes and organ maturation and development occur in pediatrics, latamoxef pharmacokinetic parameters are highly variable. Therefore, an appropriate dosing regimen is essential. Latamoxef dosing optimization in pediatrics should comprehensively consider current body weight, postnatal age, postmenstrual age, and different MIC values. Although there are several dosing recommendations for neonates, infants, and children, these recommendations are simulation results and are still considered for validation in future clinical practices. Currently, there are fewer reports of latamoxef-related adverse drug reactions in pediatrics, but some insidious aspects need to be focused on. Due to the NMTT side chain, latamoxef may generate coagulation disorders and bleeding risks, especially in the malnutritional patients, and may cause disulfiram-like reactions when co-administered with medicinal preparations containing ethanol. And for those with a penicillin allergy, latamoxef should be used with caution.
